# Supplementary figures and images for: Ethanol treatment for sterilization, concentration, and stabilization of a biodegradable plastic–degrading enzyme from Pseudozyma antarctica culture supernatant
Source: PLoS One. 2021 Jun 4;16(6):e0252811. doi: 10.1371/journal.pone.0252811 (PMC8177473; doi:10.1371/journal.pone.0252811)

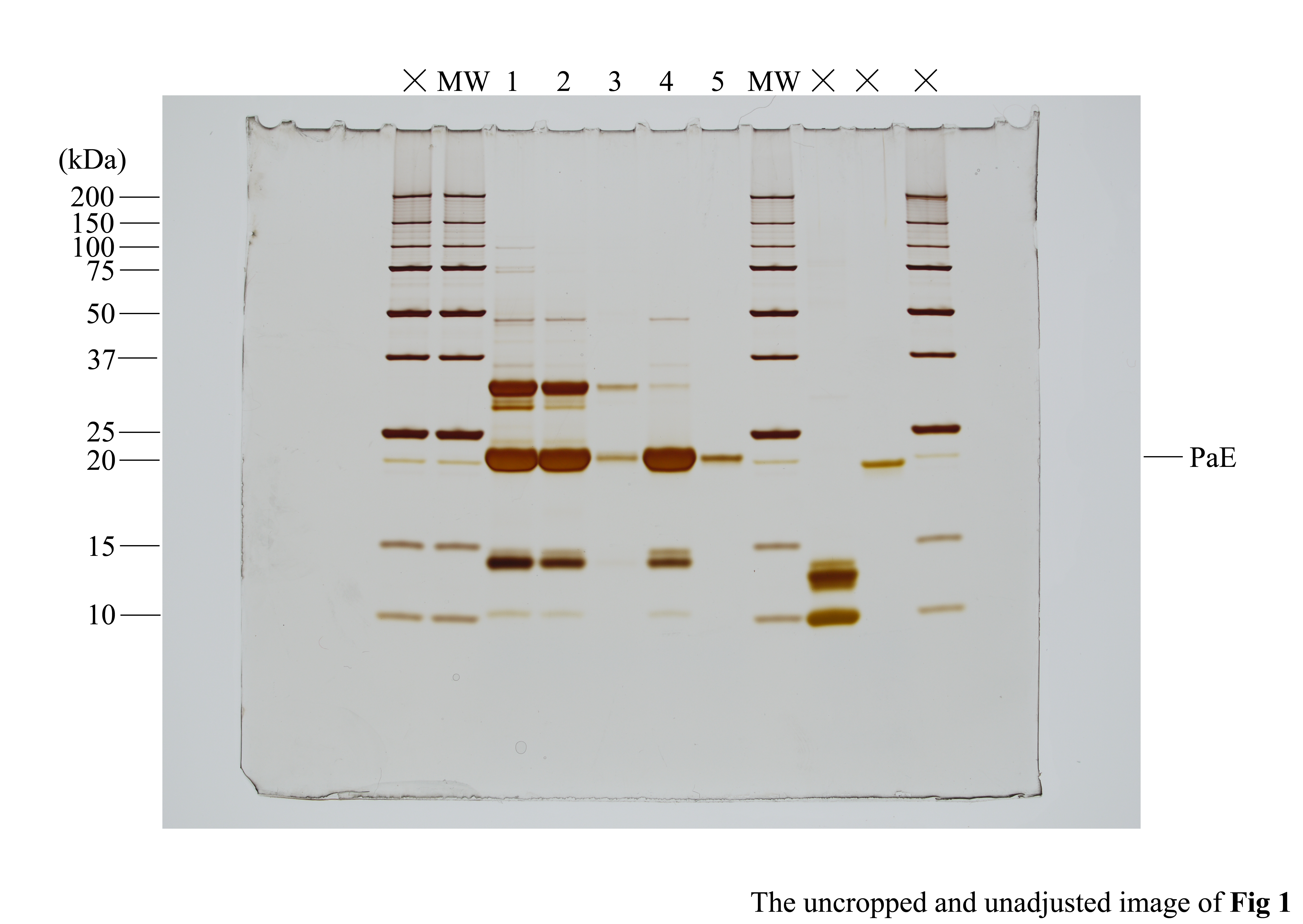

Supplement: S1 Raw image — (TIF) [file pone.0252811.s001.tif]

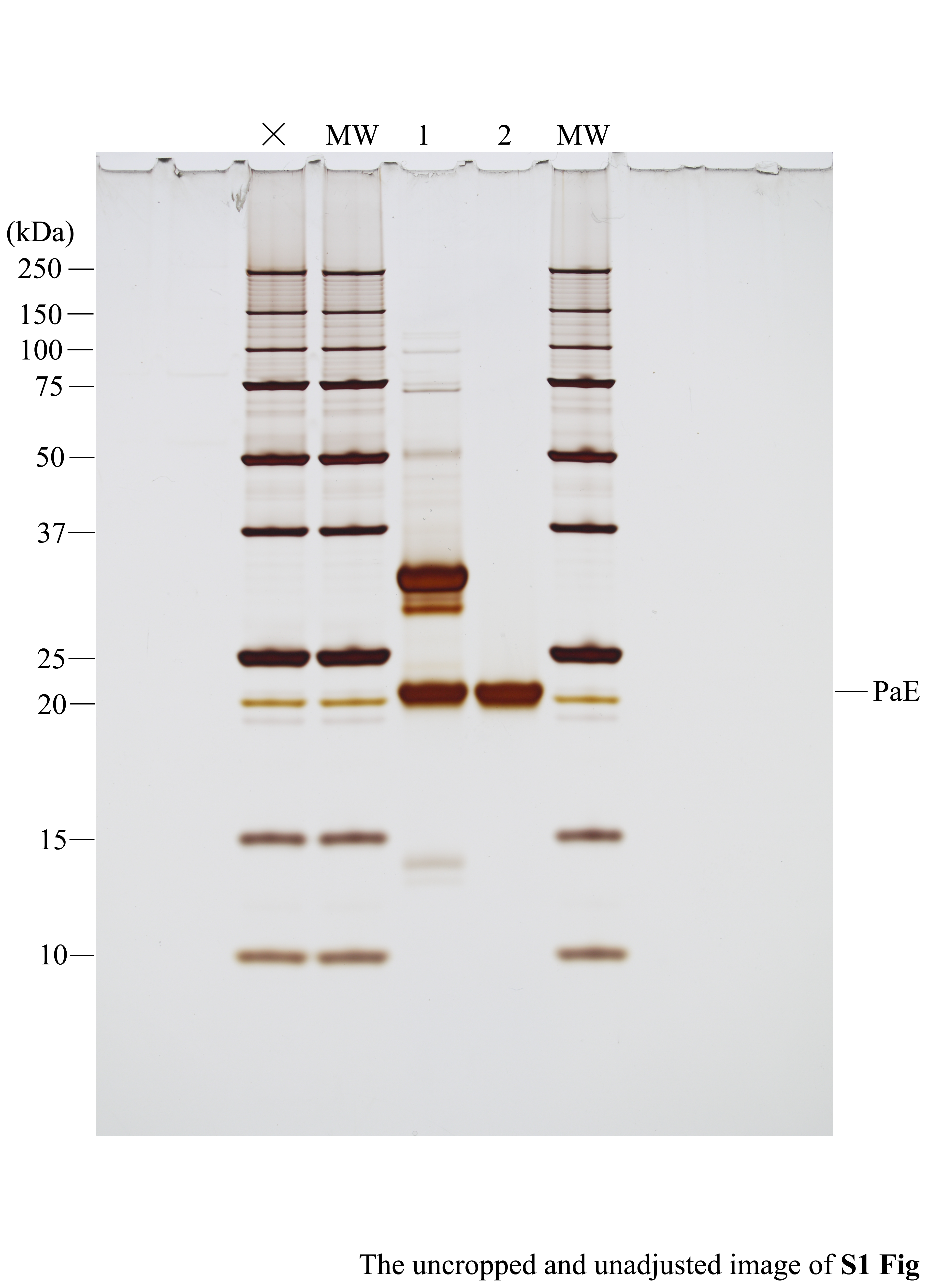

Supplement: S2 Raw image — (TIF) [file pone.0252811.s002.tif]
