# Supplementary material for: Ethanol treatment for sterilization, concentration, and stabilization of a biodegradable plastic–degrading enzyme from Pseudozyma antarctica culture supernatant
Source: PLoS One. 2021 Jun 4;16(6):e0252811. doi: 10.1371/journal.pone.0252811 (PMC8177473; doi:10.1371/journal.pone.0252811)
Supplement: S1 File — MW, molecular weight marker (Precision Plus Protein Unstained Standards, Bio-Rad Laboratories Inc.); 1, culture filtrate of Pseudozyma antarctica X-14 strain; 2, purified PaE that was eluted from a Mono S column (GE Healthcare Life Sciences) using a linear gradient of 0–0.2 M NaCl and concentrated with an Amicon Ultra Centrifugal Filter (pore size: 3 kDa; Merck KGaA). (DOCX) [file pone.0252811.s003.docx]

**Ethanol treatment for sterilization, concentration, and stabilization of a biodegradable plastic–degrading enzyme from *Pseudozyma antarctica* culture supernatant**

Takumi Tanaka^1¶^, Ken Suzuki^1¶^, Hirokazu Ueda^1^, Yuka Sameshima-Yamashita^1^, Hiroko Kitamoto^1,*^

^1^Institute for Agro-Environmental Sciences (NIAES), National Agriculture and Food Research Organization, Kannondai, Tsukuba, Ibaraki, Japan

*Corresponding author

E-mail: kitamoto@affrc.go.jp

**^¶^**These authors equally contributed to this work

**Purification of PaE from partially purified PaE**

A transformant strain with high PaE productivity, *Pseudozyma antarctica* X-14, was constructed from the wild-type strain by introducing an *Ssp*I-digested PaE-producing plasmid pPAX1-neo::PaCLE1 [1] and then stored in our laboratory. PaE was obtained from *P. antarctica* X-14 by using a jar fermentor as previously described [1].

PaE in the culture filtrate of *P. antarctica* X-14 was concentrated (hereafter “partially purified PaE”) by the combination of ammonium sulfate precipitation and ethanol precipitation, as described in the “**Materials and Methods**” section of the main article. Then, PaE was purified from the partially purified PaE as described previously [2], with modifications, as follows: The precipitate of partially purified PaE was dissolved in 20 mM 2-(*N*-morpholino)ethanesulfonic acid-NaOH buffer (pH 6.0) and applied to a Mono S column (GE Healthcare Life Sciences, Buckinghamshire, England), then eluted with a linear gradient of NaCl from 0 to 0.2 M with a flow rate of 0.25 mL min^−1^. The fractions with emulsified polybutylene succinate-*co*-adipate-degrading activity were collected and then concentrated with an Amicon Ultra Centrifugal Filter (pore size, 3 kDa; Merck KGaA, Darmstadt, Germany) while simultaneously changing the buffer to water. The purity of the isolated PaE was checked by sodium dodecyl sulfate–polyacrylamide gel electrophoresis (S1 Fig).

**References**

1. Watanabe T, Morita T, Koike H, Yarimizu T, Shinozaki Y, Sameshima-Yamashita Y, et al. High-level recombinant protein production by the basidiomycetous yeast *Pseudozyma antarctica* under a xylose-inducible xylanase promoter. Appl Microbiol Biotechnol. 2016;100:3207–3217. doi: 10.1007/s00253-015-7232-7
2. Kitamoto HK, Shinozaki Y, Cao X, Morita T, Konishi M, Tago K, et al. Phyllosphere yeasts rapidly break down biodegradable plastics. AMB Express. 2011;1:44. doi: 10.1186/2191-0855-1-44

**
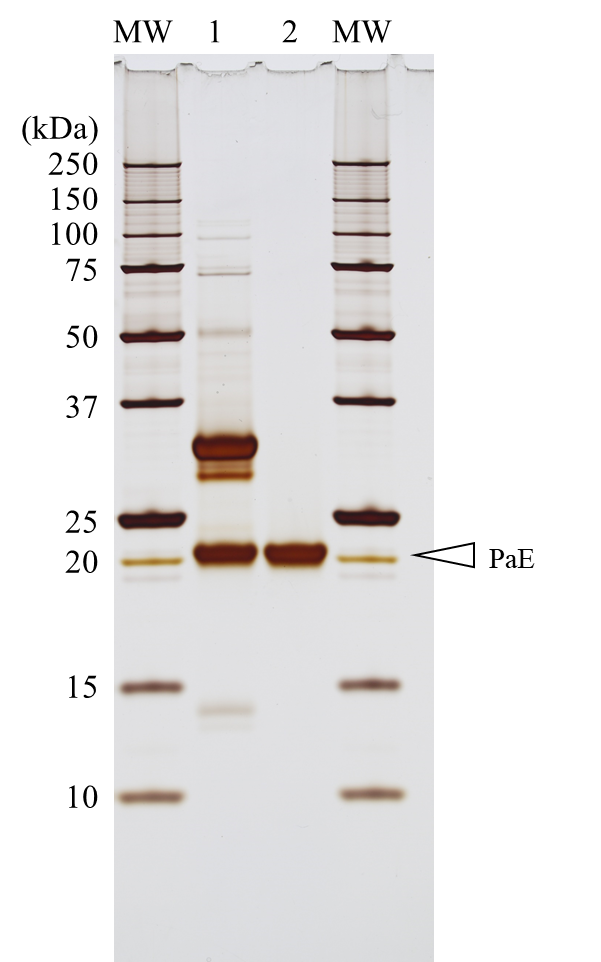
**

**S1 Fig. SDS-PAGE analysis of the purified PaE.**

MW, molecular weight marker (Precision Plus Protein Unstained Standards, Bio-Rad Laboratories Inc.); 1, culture filtrate of *Pseudozyma antarctica* X-14 strain; 2, purified PaE that was eluted from a Mono S column (GE Healthcare Life Sciences) using a linear gradient of 0 to 0.2 M NaCl and concentrated with an Amicon Ultra Centrifugal Filter (pore size: 3 kDa; Merck KGaA).
