# Supplementary material for: Ethanol treatment for sterilization, concentration, and stabilization of a biodegradable plastic–degrading enzyme from Pseudozyma antarctica culture supernatant
Source: PLoS One. 2021 Jun 4;16(6):e0252811. doi: 10.1371/journal.pone.0252811 (PMC8177473; doi:10.1371/journal.pone.0252811)
Supplement: Attachment — Submitted filename: PLos one review.docx [file pone.0252811.s004.docx]

**Comments**

The manuscript entitled “Ethanol treatment for sterilization, concentration, and stabilization of biodegradable plastic–degrading enzyme from *Pseudozyma antarctica* culture supernatant” is well- organized with good clarity.

The authors have proposed a new combinatorial method to sterilize, precipitate and to increase the shelf life of PaE enzyme. The authors state that PaE kept more than 60% of its PBSA-degrading activity 20% (v/v) ethanol solution at room temperature for more than 7 months and thus claims that with this method a large volume of PaE solution can be stored without any special equipment.

It is preferable that the discussion section should include the comparative analysis of the specific activity and yield of the enzyme precipitated by the usual method and this method, in order to highlight the advantage / importance of this research work. The entire manuscript talks about this research study but it is good to compare the work with that of previous reports, so that it would give a more clear idea for the readers.

I conclude that minor language polishing is required and recommend acceptance of the manuscript with minor revision.
