# Supplementary material for: Ethanol treatment for sterilization, concentration, and stabilization of a biodegradable plastic–degrading enzyme from Pseudozyma antarctica culture supernatant
Source: PLoS One. 2021 Jun 4;16(6):e0252811. doi: 10.1371/journal.pone.0252811 (PMC8177473; doi:10.1371/journal.pone.0252811)
Supplement: Attachment — Submitted filename: Response to Reviewers.docx [file pone.0252811.s005.docx]

Dear Editor of PLOS ONE,

We are writing to resubmit the revised version of our manuscript entitled “**Ethanol treatment for sterilization, concentration, and stabilization of a biodegradable plastic–degrading enzyme from *Pseudozyma antarctica* culture supernatant.**” (PONE-D-20-38928) for consideration of publication as a **Research Article** in PLOS ONE. The revised manuscript contains 21 pages of text, 3 tables, 2 figures, 3 supplemental material, all of which have been prepared according to the instructions provided by PLOS ONE. The English in this manuscript was carefully corrected by two native-English speaking, professional editors, both with extensive research editing experience.

We would like to thank the reviewers and yourself for the detailed comments, which have helped us to improve our manuscript.

We have carefully read both the editor’s and reviewers’ comments and have revised the manuscript accordingly. Below you will find our point-by-point responses to each of the comments. We have modified the manuscript according to the reviewers’ suggestions. All changes are highlighted in yellow.

We hope that our revisions are acceptable and that our manuscript is now suitable for publication in PLOS ONE.

Sincerely yours,

Hiroko Kitamoto, Ph.D.

Environmental Biofunction Unit, Division of Biogeochemical Cycles, Institute for Agro-Environmental Sciences, National Agriculture and Food Research Organization

3-1-3 Kannondai, Tsukuba, Ibaraki 305-8604, Japan

TEL: +81-29-838-8356, E-mail: kitamoto@affrc.go.jp

**[Response to Reviewer #1]**

The overall study seems interesting, however, few queries needs to be addressed.

→ We thank the reviewers for their helpful comments and suggestions. Below are our point-by-point responses to the reviewer’s comments.

1. The grammar is poor. The manuscript needs extensively revised.

→ We have modified the entire manuscript. The manuscript has been edited carefully by two native English-speaking professional editors from ELSS, Inc. (elss@elss.co.jp, http://www.elss.co.jp). All changes are highlighted in yellow.

2. Reframe the abstract.

→ We have edited the abstract to reflect the content of the manuscript more accurately as follows:

Line nos. 24–42 (revised manuscript):

Biodegradable plastics must be sufficiently stable to maintain functionality during use but need to be able to degrade rapidly after use. We previously reported that treatment with an enzyme named PaE, secreted by the basidiomycete *yeast Pseudozyma antarctica* can speed up this degradation. To facilitate the production of large quantities of PaE, here, we aimed to elucidate the optimal conditions of ethanol treatment for sterilization of the culture supernatant and for concentration and stabilization of PaE.

The results showed that *Pseudozyma antarctica* completely lost its proliferating ability when incubated in ≥20% (v/v) ethanol. When the ethanol concentration was raised to 90% (v/v), PaE formed a precipitate; however, its activity was restored completely when the precipitate was dissolved in water. To reduce ethanol use, PaE was successfully concentrated and recovered by sequential ammonium sulfate precipitation and ethanol precipitation steps. Over 90% of the activity in the original culture supernatant was recovered and the specific activity was increased 3.4-fold. By preparing the enzyme solution at a final concentration of 20% (v/v) ethanol, about 60% of the initial activity was maintained at ambient temperature for over 6 months without growth of microbes. We conclude that ethanol treatment is effective for sterilization, concentration, and stabilization of PaE, and that concentrating PaE by sequential ammonium sulfate precipitation and ethanol.

3. Correct line no. 50

→ We have corrected this sentence, and have added references.

Line nos. 45–47 (revised manuscript):

Since large-scale production of the plastics started in the 1950’s, more than 8 billion tons of plastic products have been used worldwide, and more than 6 billion tons have been disposed of as waste [1,2].

In addition, we have edited the Introduction (see changes highlighted in yellow in the manuscript file)

4. Edit line no. 130

→ We have modified this sentence to describe in more detail how the concentration of purified PaE was calculated:

Line nos. 131–139 (revised manuscript):

The concentration of purified PaE was calculated as described previously [13]. In short, the purified PaE solution was appropriately diluted, and its absorbance was measured at 280 nm (A_280_). Then, the concentration of PaE was calculated according to the following equation: A_280_ (*M*^−1^ cm^−1^) = 5,500n_W_ + 1,490n_Y_ + 125n_C_ [17,18], where *M* (=20,362.41) is the average molecular weight calculated using GENETYX software ver. 9 (GENETYX, Tokyo, Japan), n_W_ is the number of Trp residues (=1), n_Y_ is the number of Tyr residues (=7), and n_C_ is the number of disulfide bonds (=2), per polypeptide chain based on the mature amino acid sequence of PaE [6].

5. Edit line no. 269

→ We have edited line no 269 and have added a new paragraph above it as follows:

Line nos. 278–284 (revised manuscript):

When PaE is used for BP product degradation, it is desirable to be able to sterilize the culture media after enzyme production, concentrate the enzyme from a large amount of the culture supernatant, and both stabilize and store the enzyme for a long period of time. Here, we explored the possibility of using ethanol treatment to achieve these objectives and investigated the optimal conditions.

First, we examined the degree of inactivation of *P. antarctica* cells following treatment with various concentrations of ethanol.

In addition, we have edited the Discussion (see changes highlighted in yellow in the manuscript file)

6. Edit line no. 282

→ We have edited line no. 282 and its surrounding lines as follows:

Line nos. 299–302 (revised manuscript):

After storage at ambient temperature for 206 days, the PaE activity of the samples with ethanol concentrations of 10% or 25% remained at 67% or 60% of the initial value, respectively, which was higher than the activity remaining at higher ethanol concentrations (Fig 2a).

7. Cite some more references

→ We have cited eight new references. In addition, we have corrected the reference style to match the style and format of PLOS ONE.

**[Response to Reviewer #2]**

It is preferable that the discussion section should include the comparative analysis of the specific activity and yield of the enzyme precipitated by the usual method and this method, in order to highlight the advantage / importance of this research work. The entire manuscript talks about this research study but it is good to compare the work with that of previous reports, so that it would give a more clear idea for the readers.

→ We thank the reviewers for their helpful comment and the suggestion. We tried to compare this work with that of previous reports. We previously reported purification of PaE from the culture filtrate of *Pseudozyma antarctica* (Kitamoto et al, 2011; reference no. 12 in the revised manuscript). In this report, enzyme-producing microbe can be removed from the culture supernatant by passage through a cell impermeable membrane using a dedicated facility, however, the concentration and preservation steps are still necessary. Therefore, we have added a new paragraph and new a sentence in the Discussion as follows:

Line nos. 278–282 (revised manuscript):

When PaE is used for BP product degradation, it is desirable to be able to sterilize the culture media after enzyme production, concentrate the enzyme from a large amount of the culture supernatant, and both stabilize and store the enzyme for a long period of time. Here, we explored the possibility of using ethanol treatment to achieve these objectives and investigated the optimal conditions.

Line nos. 334–335 (revised manuscript):

The ethanol treatment serves all the requirements for the preparation of enzyme.
